# Quantum Yield Enhancement in Graphene Quantum Dots via Esterification with Benzyl Alcohol

**DOI:** 10.1038/s41598-019-50666-3

**Published:** 2019-10-01

**Authors:** Suzuka Tachi, Hiroki Morita, Misaki Takahashi, Yusuke Okabayashi, Takuya Hosokai, Toshiki Sugai, Shota Kuwahara

**Affiliations:** 1 Department of Chemistry, Faculty of Science, Toho University, 2-2-1 Miyama, Funabashi, Chiba 274-8510 Japan; 2 National Institute of Advanced Industrial Science and Technology (AIST), 1-1-1 Umezono, Tsukuba, Ibaraki 305-8568 Japan

**Keywords:** Nanoparticles, Quantum dots, Nanoparticles

## Abstract

The quantum yield of graphene quantum dots was enhanced by restriction of the rotation and vibration of surface functional groups on the edges of the graphene quantum dots via esterification with benzyl alcohol; this enhancement is crucial for the widespread application of graphene quantum dots in light-harvesting devices and optoelectronics. The obtained graphene quantum dots with highly graphene-stacked structures are understood to participate in π–π interactions with adjacent aromatic rings of the benzylic ester on the edges of the graphene quantum dots, thus impeding the nonradiative recombination process in graphene quantum dots. Furthermore, the crude graphene quantum dots were in a gel-like solid form and showed white luminescence under blue light illumination. Our results show the potential for improving the photophysical properties of nanomaterials, such as the quantum yield and band-gap energy for emission, by controlling the functional groups on the surface of graphene quantum dots through an organic modification approach.

## Introduction

Graphene quantum dots (GQDs), carbon-based nanometre-sized materials, have a size-dependent photophysical property originating from the quantum confinement effect; many applications have been proposed, such as bioimaging probes, sensors, light emitting devices and solar cells^1–8^. However, the relatively low quantum yields (QYs) of GQDs (approximately 0.10)^3,8–10^ compared to those of commercially available organic compounds and semiconductor-based quantum dots limit the general use of GQDs as luminescent materials. Previous papers have revealed that the fluorescence of GQDs occurs by defect state emission and/or intrinsic state emission^9,11^. S. Zhu and co-workers reported that the blue emission of GQDs (<450 nm) was intrinsic state emission, while the green emission (>500 nm) was defect state emission^11^. These authors also confirmed that reduction or other modification of the initial epoxy and carboxyl groups on GQDs, which induced nonradiative recombination of localized electron-hole pairs, resulted in the promotion of intrinsic state emission of blue luminescence because OH groups suppressed the nonradiative emission and defect state emission due to the reduction in defects in GQDs. The doping of heteroatoms, e.g., nitrogen, into GQDs is also quite effective at enhancing the QYs, similar to the case of carbon dots, and this method is the recent trend for obtaining GQDs with high QYs^12,13^.

In this study, we suppressed the flexibility arising from vibration and rotation of the surface functional groups on the edges of GQDs by replacing the carboxyl groups with aromatic ester groups. In the case of gold clusters, a well-known fluorescent material, it was reported that suppression of the intramolecular vibration and rotation greatly increased the QYs of the gold clusters^14,15^, demonstrating that this method is an acceptable way to increase the QYs of GQDs via a chemical edge modification. We synthesized esterified GQDs with benzyl alcohol, a simple aromatic alcohol compared to the previously reported compounds for the edge modification of GQDs^16–18^, which exhibited a stacked structure of graphene sheets with an average diameter of 24.7 nm, and the absolute QYs reached to 0.25 in toluene. The obtained esterified GQDs, which originally existed in a gel-like solid form, were partially soluble in water, and emitted blue fluorescence. Furthermore, we found that the obtained gel-like solid esterified GQDs emitted white fluorescence similar to the edge-functionalized GQDs with dendritic wedges^17^.

## Results and Discussion

### Structural characterization

**Figure 1:**
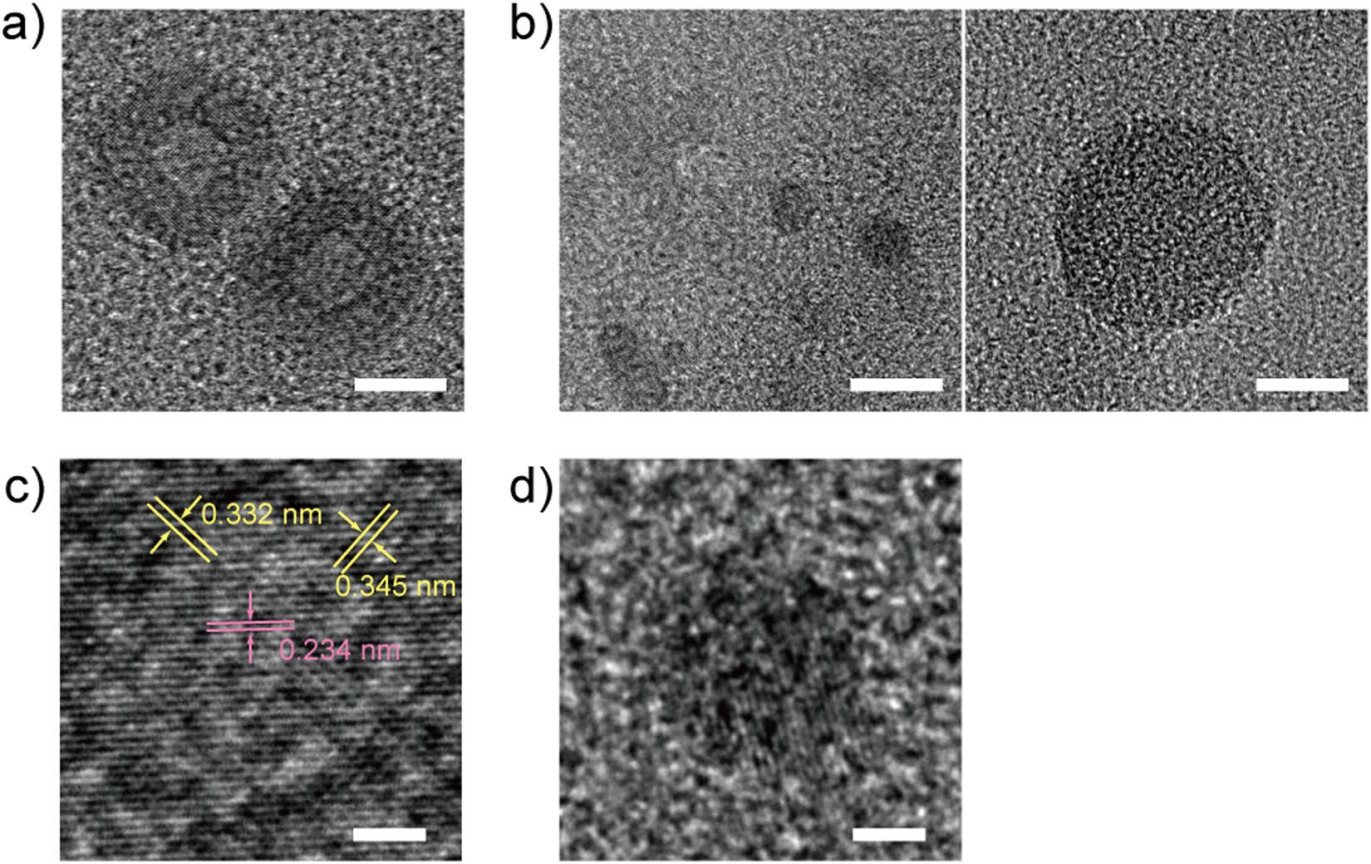
TEM images of (**a**) GQD-Bn (O) and (**b**) GQD-Bn (W) (scale bar: 10 nm); the corresponding HRTEM images of (**c**) GQD-Bn (O) and (**d**) GQD-Bn (W) (scale bar: 2 nm).

**Figure 2:**
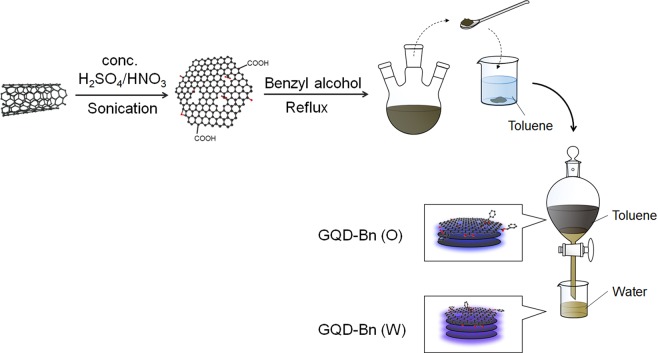
Scheme representing the synthesis of GQD-Bn.

The structure of the GQDs esterified with benzyl alcohol (GQD-Bn) was investigated by using high-resolution transmission electron microscopy (HRTEM), as shown in Fig. 1. The average diameter of GQD-Bn extracted into toluene (GQD-Bn (O) in Fig. 2) was 24.7 ± 10.0 nm (see Supplementary Fig. S1), whereas that of GQD-Bn remaining in water (GQD-Bn (W)) was 4.8 ± 1.4 nm (see Supplementary Fig. S1). The size distribution of GQD-Bn (W) showed another peak at an average diameter of 20.1 ± 6.0 nm for a large round thin layer, which was counted at the same rate in HRTEM observations. A crossed lattice pattern with a lattice parameter of 0.327 ± 0.005 nm, corresponding to the (002) interlayer spacing and similar to the value of bulk graphite^9^, was observed in the HRTEM image of GQD-Bn (O), indicating the high crystallinity of the obtained GQDs. In addition, 12% of the observed individual GQD-Bn (O) (32 GQDs counted) also showed a lattice pattern with an interlayer spacing of 0.245 ± 0.010 nm, corresponding to the (1120) lattice fringe of graphene^19,20^ in addition to the (002) lattice patterns (Fig. 1c). The lattice patterns observed in GQD-Bn (O) indicate that GQD-Bn (O) has a highly graphene-stacked structure. In the case of GQD-Bn (W), a lattice parameter of 0.256 ± 0.023 nm was observed for the small particle, whereas no patterns appeared for the large round thin layer (Fig. 1b,d). The esterification of GQDs with benzyl alcohol seems to result in formation of a highly stacked structure of large, round GQD layers that are soluble in toluene. GQDs with a small particle structure and a large thin layer were observed in water, which suggests that hydrophilic functional groups such as hydroxyl and carboxyl groups remain in the GQD-Bn (W) due to insufficient esterification within their structures. When 1-hexanol or 1-decanol—aliphatic ester—was used for the esterification instead of benzyl alcohol, the obtained GQDs (GQD-Hex and GQD-Dec, respectively) had a small particle shape with a diameter of approximately 5 nm, which is the same as that of GQD-Bn (W). Note that the size distributions of GQD-Hex and GQD-Dec do not contain the large GQDs, which are understood to remain in water when the esterified GQDs were extracted into toluene due to insufficient functionalization to be soluble in an organic solvent.

**Figure 3:**
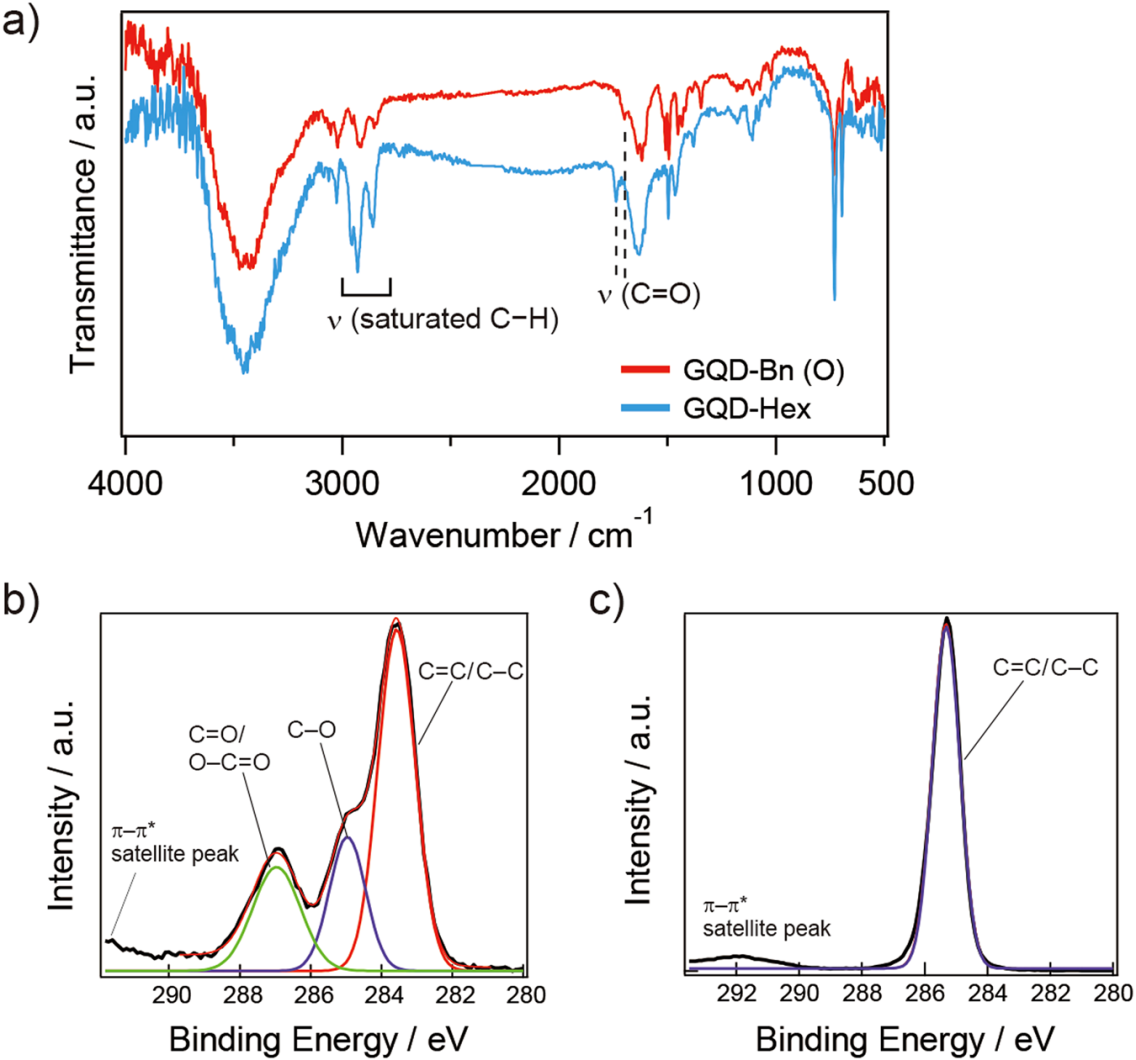
(**a**) FTIR spectra of GQD-Bn (O) (red) and GQD-Hex (blue). High-resolution XPS spectra of C1s for **(b**) before and **(c**) after esterification of GQDs with benzyl alcohol (GQD-Bn (O)). Each π–π* satellite peak at 292 eV is used as a reference for charge correction.

As shown in Fig. 3a, the esterification of GQD-Bn (O) was confirmed by Fourier transform infrared (FTIR) spectra from the peak shift of the C=O stretching mode observed at 1698 cm^−1^ for GQD-Bn (O), which is different from the C=O stretching mode observed for water-soluble GQDs, as previously reported (~1720 cm^−1^)^9,16,21^. In the case of GQD-Hex, peaks related to the vibration modes of the ester group and the saturated C–H moieties were observed at 1740 cm^−1^ and 2920 cm^−1^, respectively, in the FTIR spectra and are attributed to the resultant aliphatic ester with 1-hexanol. These FTIR spectra confirm that the esterification of GQDs was successfully completed via the chemical reaction performed in this work. The high-resolution X-ray photoelectron spectroscopy (XPS) spectra shown in Fig. 3b,c confirm the modification of the functional groups in GQDs via esterification with benzyl alcohol because of the absence of a C–O peak (285 eV) and a C=O/O–C=O peak (287 eV)^16^. Note that GQD-Bn (O) on a fluorine-doped tin oxide substrate for the XPS measurement showed film like form, which seems to affect the detection of XPS signal related to ester groups on the edges of the GQDs. The Raman spectra of GQD-Bn could not be obtained at a wavelength of 780 nm due to a strong photoluminescence background of the GQD-Bn itself.

### Optical characterization

**Figure 4:**
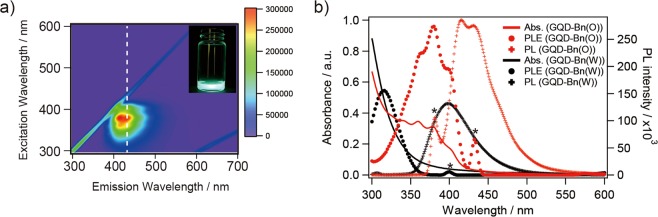
(**a**) PL excitation map for GQD-Bn (O). The dotted line indicates the wavelength at which the cross-sectional PL excitation spectrum shown in (**b**) is obtained. (inset) Photographs of GQD-Bn (O) taken under UV illumination (λ = 410 nm). The colour indicates the intensity of the emission normalized at each emission peak. (**b**) Optical absorption spectra (solid line), PL excitation (PLE) (circle) and PL emission spectra (cross) of GQD-Bn (O) (red) and GQD-Bn (W) (black); the emission wavelengths for PLE are 431 nm and 400 nm, and the excitation wavelengths for PL emission spectra are 380 nm and 330 nm, respectively. The asterisks shown on the PLE and the PL emission spectra indicate the peaks derived from Rayleigh scattering of the excitation light.

Figure 4a shows the excitation and emission contour maps of fluorescence intensity for the GQD-Bn (O) sample. The emission maximum was 416 nm for GQD-Bn (O) and was independent of the excitation wavelength, indicating that the photoluminescence (PL) of GQD-Bn (O) was attributed to intrinsic state emission (electron-hole recombination, quantum size effect/zig-zag sites)^11^. The PL excitation spectrum of GQD-Bn (O) was well correlated with the absorption spectrum, with the characteristic feature observed in the range of 350–400 nm (Fig. 4b) as arising from a transition from the highest occupied molecular orbital (π-orbital) to the lowest unoccupied molecular orbital^3,9^. As summarized in Table 1, all esterified GQDs showed blue fluorescence ascribed to intrinsic state emission. When aliphatic alcohols were used for esterification instead of benzyl alcohol, the emission wavelength was blueshifted to 400–406 nm with a decrease in the size of GQDs, which demonstrates that the obtained GQDs behaved as quantum dots (see Supplementary Fig. S3).

**Table 1:** The structure and PL properties of esterified GQDs synthesized from different types of alcohol.

	Diameter/nm	λ_ex,max_^[a]^/nm	λ_em,max_^[b]^/nm	Φ/Φ_Bn(O)_^[c]^	τ_1_/ns	τ_2_/ns	C_2_/C_1_^[d]^	τ_avg_^[e]^/ns
GQD-Bn (O)	24.7 ± 10.0	380	416	1.0	1.66 ± 0.04	7.24 ± 0.14	0.96	6.2
GQD-Bn (W)	4.8 ± 1.4 20.1 ± 6.0	316	400	0.65	1.97 ± 0.12	8.34 ± 0.05	6.2	8.1
GQD-Hex	3.2 ± 1.3	340	406	0.15	1.62 ± 0.03	5.55 ± 0.15	0.42	3.9
GQD-Dec	5.4 ± 1.4	340	396	0.37	1.55 ± 0.12	5.85 ± 0.23	0.24	3.6
GQD (before esterification)		330	532	0.008				
GQD-Bn (O) ^[f]^		380	431	0.81				

^[a]^Excitation wavelength with maximum intensity. ^[a]^Emission wavelength with maximum intensity. ^[c]^Relative QY calculated by the function Φ_i_/Φ_Bn(O)_  = (A_st_/A_i_)·(F_i_/F_st_)·(n_i_^2^/n_st_^2^)·(D_i_/D_st_), where i is the sample, st is the reference, A is the absorbance at the excitation wavelength at which the PL spectra are measured, F is the peak area, n is the refractive index, and D is the dilution rate. GQD-Bn (O) in toluene was used as the reference for the calculation. A_i_ and F_i_ are calculated by using the absorbance at λ_ex, max_ and the peak area of the PL spectrum excited at λ_ex, max_, respectively, of each sample. ^[d]^The intensity ratio of the slow decay component (C_2_) to the fast decay component (C_1_). ^[e]^τ_avg_ calculated by the function τ_avg_ = Σ(C·τ^2^)/Σ(C·τ) = (C_1_·τ_1_^2^ + C_2_·τ_2_^2^)/(C_1_·τ_1_ + C_2_·τ_2_). ^[f]^Graphite was used as the starting material for synthesizing GQD-Bn (O).

The absolute QY of GQD-Bn (O) in toluene was 0.25 at a wavelength of 400 nm, and the relative QY of GQD-Bn (O) was 3–5 times higher than those of GQD-Hex and GQD-Dec (see Table 1). In addition, as seen by comparing the relative QY of GQD-Bn (O) with those of non-functionalized GQDs, the QY of GQD-Bn (O) was approximately 100 times higher than those of non-functionalized GQDs. The highly stacked structure in GQD-Bn (O) leads to π–π interactions with adjacent aromatic rings of the benzylic ester on the edges of the GQDs, decreasing the flexibility arising from vibration and rotation of the surface functional groups and thus impeding the nonradiative recombination process and increasing the QY of GQDs. Although the doped GQDs still have higher QYs than GQDs with rigid functional groups^22^, our approach to utilize π–π interactions to impede the nonradiative recombination process in GQDs efficiently improves the QY of GQDs. Note that the same QY enhancement was observed when graphite was used as the starting material instead of carbon nanotubes^23^ (see Table 1 and Supplementary Fig. S4).

**Figure 5:**
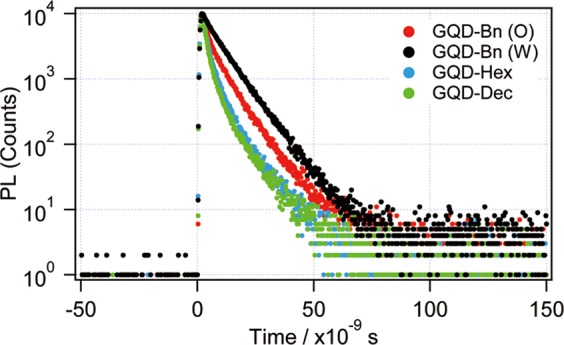
PL decay traces of GQD-Bn (O) (red), GQD-Bn (W) (black), GQD-Hex (blue) and GQD-Dec (green). The vertical axis is plotted on a logarithmic scale.

Figure 5 shows the time-resolved PL decay profiles of the esterified GQDs, and the corresponding lifetimes, obtained by fitting simulations using exponential decay functions, are also summarized in Table 1. The obtained PL decay consisted of two components^24^, and the PL decay time of the fast decay component was τ_1_ = ~2 ns, regardless of the type of ester groups, whereas that of the slow decay component (τ_2_ = 5–8 ns), and the intensity ratio of the slow decay component to the fast decay component (C_2_/C_1_) depended on the type of ester group. The C_2_/C_1_ values of GQDs formed with benzylic ester were higher than those of GQDs with an aliphatic ester, corresponding to the Φ/Φ_Bn(O)_ value in Table 1. The two relaxation paths are understood to exist in the esterified GQDs; both a high probability and a long decay time following the slow relaxation path are understood to increase the QYs of GQDs with the aromatic ester. GQD-Bn (W) with the highest C_2_/C_1_, however, showed slightly lower QYs than GQD-Bn (O), suggesting that several factors, such as the polarity of the solvent, hydrogen bonding and nonradiative recombination due to the remaining carboxyl and epoxy groups, affect both of the relaxation path of excited carriers and the QYs in water.

### Solid-state GQD-Bn

**Figure 6:**
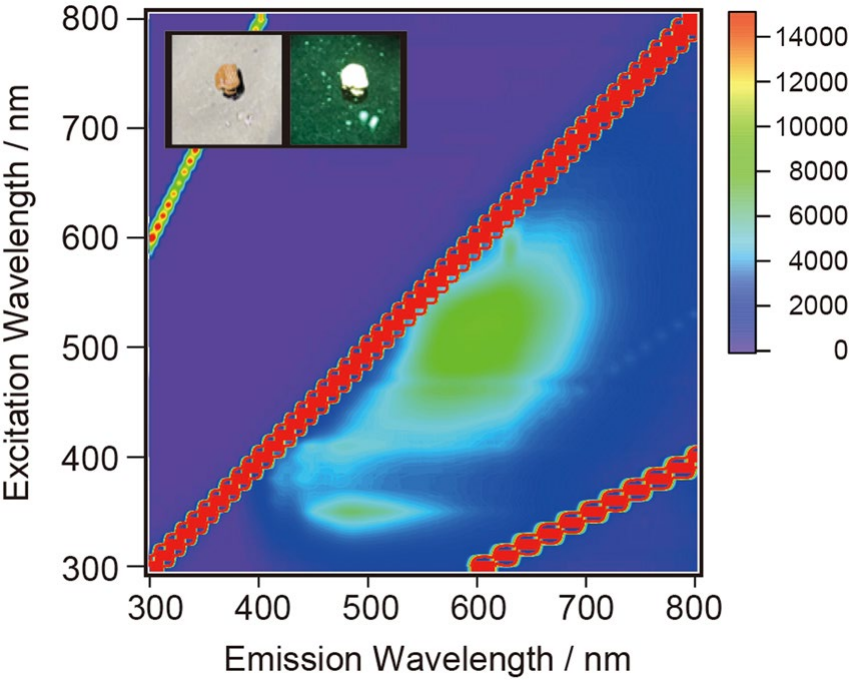
PL excitation map for solid-state GQD-Bn. The colour indicates the intensity of the emission. The red stripe of high intensity is due to Rayleigh scattering of the excitation light. The inset shows photographs of the solid-state GQD-Bn taken under white light and UV illumination (λ = 410 nm).

The crude GQD-Bn in the brown gel-like solid form showed white luminescence under UV illumination at a wavelength of 410 nm. The contour map of the fluorescence intensity for as-synthesized solid GQD-Bn is shown in Fig. 6, and a new broad emission peak appeared at approximately 600 nm. The new PL peak seems to arise from redshifted excimer-like emission^25,26^ due to the form of assembled GQDs in the gel; the nonuniform structure and ester moiety of GQD-Bn would cause the heterogeneity of interactions in neighbouring GQD-Bn, resulting in broadening of the emission feature. The time-resolved PL decay in the solid state (see Supplementary Fig. S5) was not well-fitted by using a double exponential function, indicating that GQD-Bn in the solid state includes GQDs with various PL decay times and/or complicated relaxation paths.

## Methods

### Synthesis of esterified GQDs

A 25 mg aliquot of single-walled carbon nanotubes (SWCNTs; Signis® CG100, Sigma-Aldrich, USA; diameter: 0.7–1.3 nm) was sonicated in 15 mL concentrated sulfuric acid and nitric acid (3:1) in an ultrasonic bath sonicator for 20 h at 40 °C. The obtained GQD aqueous solution was then bubbled in N_2_ gas for 10 min. Before the subsequent esterification process, 40 mL benzyl alcohol with bubbling in a N_2_ atmosphere was cooled in a 3-necked flask in an ice bath. The obtained GQD solution was gradually added to the flask, followed by refluxing in N_2_ for 3 h at 120 °C. The obtained esterified GQDs with benzyl alcohol were in a gel form and partially dissolved in water. After neutralization of the esterified GQDs in water with NaHCO_3_, the organic-soluble GQDs were extracted into toluene. The GQDs in toluene were then dehydrated by the addition of MgSO_4_, following the removal of the resulting salt by filtration.

### Structural and optical characterization of GQDs

HRTEM images were obtained on an electron microscope at 120 kV (Tecnai G2 F20 S-TWIN, FEI, Thermo Fisher Scientific, USA). Infrared spectra were obtained on an FT-IR spectrophotometer (FT/IR-4100, JASCO, Japan). X-ray photoelectron spectroscopy was carried out on an AXIS Nova (KRATOS ANALYTICAL, SHIMADZU Corp., Japan). Optical absorption spectra of the obtained samples were collected on a UV-vis-NIR spectrophotometer (UV-3600, SHIMADZU corp., Japan). All fluorescence spectra including excitation and emission contour maps were obtained by a spectrofluorophotometer (RF-6000, SHIMADZU Corp., Japan). The fluorescence lifetime was counted on a time-correlated single-photon-counting spectrometer (HORIBA, Japan) with excitation at 342 nm and a pulse width of 1.1 ns. The photoluminescence quantum efficiency was measured using an absolute photoluminescence quantum yield measurement system (C9920-02, Hamamatsu Photonics).

## Conclusion

In conclusion, the QY of GQDs was successfully enhanced by restricting the rotation and vibration of surface functional groups on the edges of GQDs via esterification with benzyl alcohol. The obtained GQDs had a highly graphene-stacked structure with high crystallinity. GQD-Bn in toluene had an absolute QY value of up to 25%, whereas GQDs with aliphatic ester groups had QYs that were 1/3–1/5 lower. The type of ester groups on GQDs affected the relaxation path for the emission of GQDs, and the slower relaxation component of PL decay preferentially occurred in GQD-Bn. The as-synthesized GQD-Bn in a gel-like form showed white luminescence with two emission regions, 400–550 nm and 550–700 nm, which is expected to have promising applications in light-harvesting devices and optoelectronics.

## Supplementary information

Supplementary Information
