# Supplementary material for: Quantum Yield Enhancement in Graphene Quantum Dots via Esterification with Benzyl Alcohol
Source: Sci Rep. 2019 Oct 1;9:14115. doi: 10.1038/s41598-019-50666-3 (PMC6773719; doi:10.1038/s41598-019-50666-3)
Supplement: Supplementary file 1 — Supplementary Information [file 41598_2019_50666_MOESM1_ESM.pdf]

Supplementary Information for

Quantum Yield Enhancement in Graphene Quantum Dots via Esterification with Benzyl Alcohol

Suzuka Tachi,[a] Hiroki Morita,[a] Misaki Takahashi,[a] Yusuke Okabayashi,[b] Takuya Hosokai,[b] Toshiki Sugai [a] and Shota Kuwahara*[a]

[a] Department of Chemistry, Faculty of Science, Toho University, 2-2-1 Miyama, Funabashi, Chiba 274-8510, Japan

[b] National Institute of Advanced Industrial Science and Technology (AIST), 1-1-1 Umezono, Tsukuba, Ibaraki 305-8568, Japan

Diameter distribution of esterified GQDs.

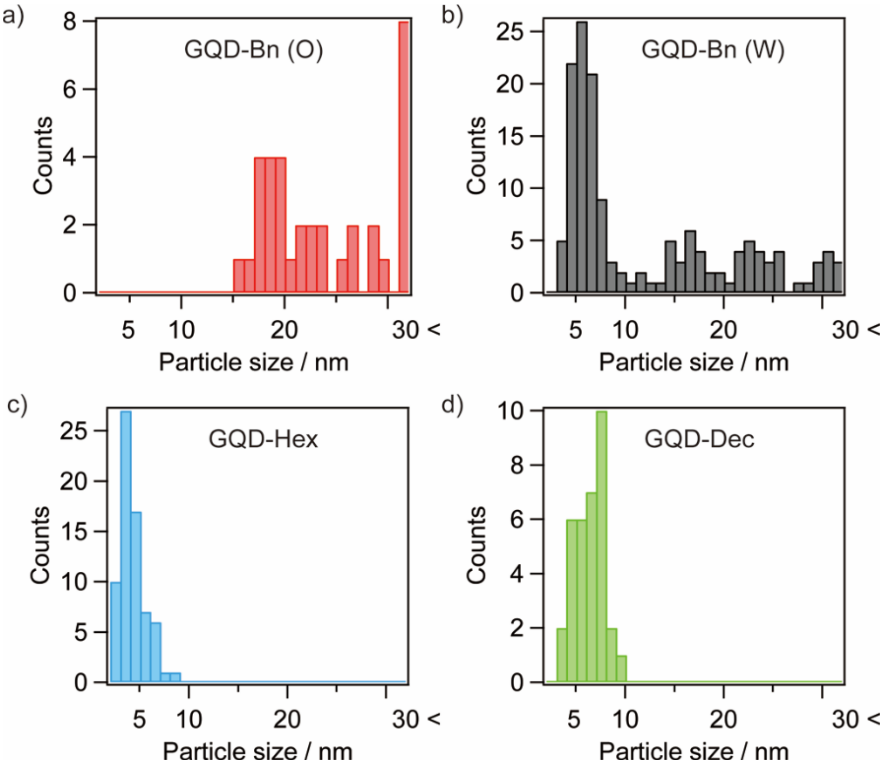

Figure S1. Diameter distribution of the esterified GQDs a) GQD-Bn (O), b) GQD-Bn (W), c) GQD-Hex and d) GQD-Dec. The far-right bar is the counts of GQDs having the diameter of more than 30 nm.

TEM images of GQD-Hex and GQD-Dec.

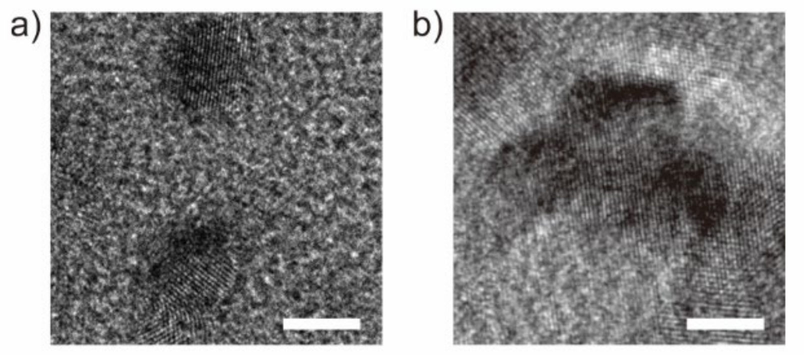

Figure S2. TEM image of a) GQD-Hex and b) GQD-Dec (Scale bar: 5 nm).

PL excitation maps of GQD before esterification, GQD-Bn (W), GQD-Hex and GQD-Dec.

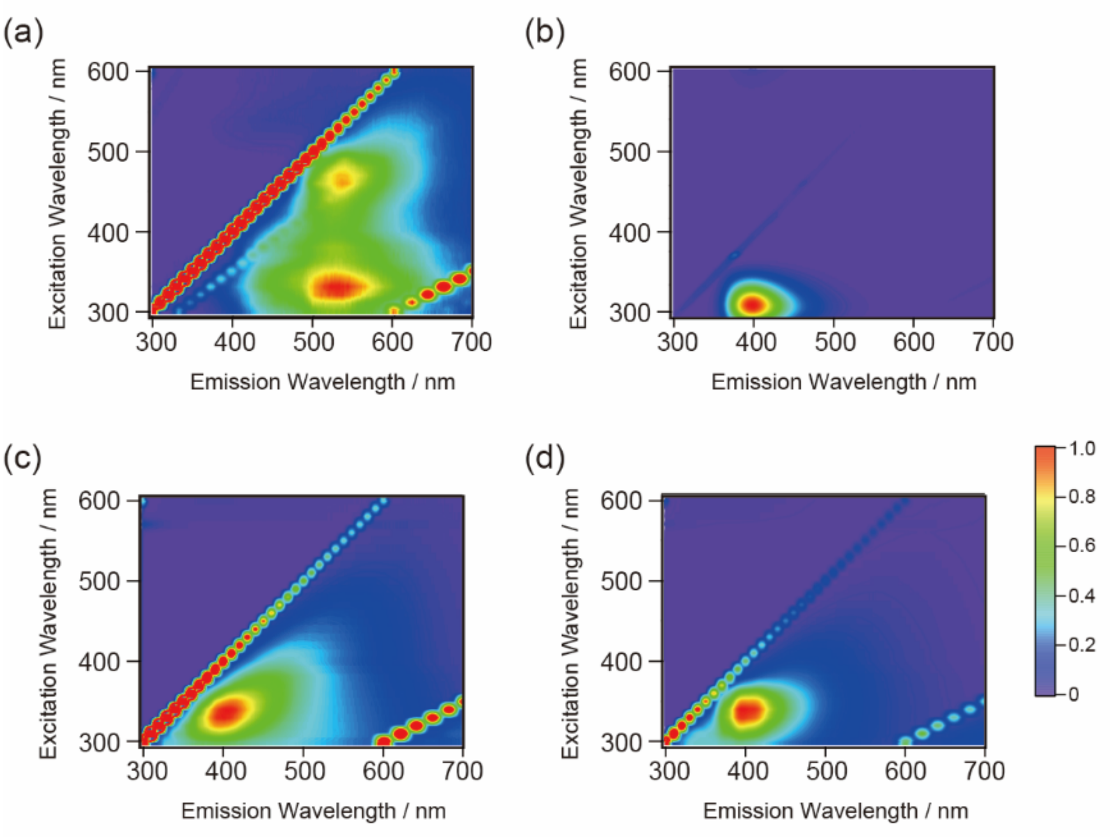

Figure S3. PL excitation map for a) GQD before esterification in water, b) GQD-Bn (W) in water, c) GQD-Hex and d) GQD-Dec in toluene, respectively. The colour indicates the intensity of the emission normalized at each emission peak.

Optical absorption spectrum, PL excitation spectra and PL excitation maps of GQD-Bn (O) synthesized by using graphite as the starting material.

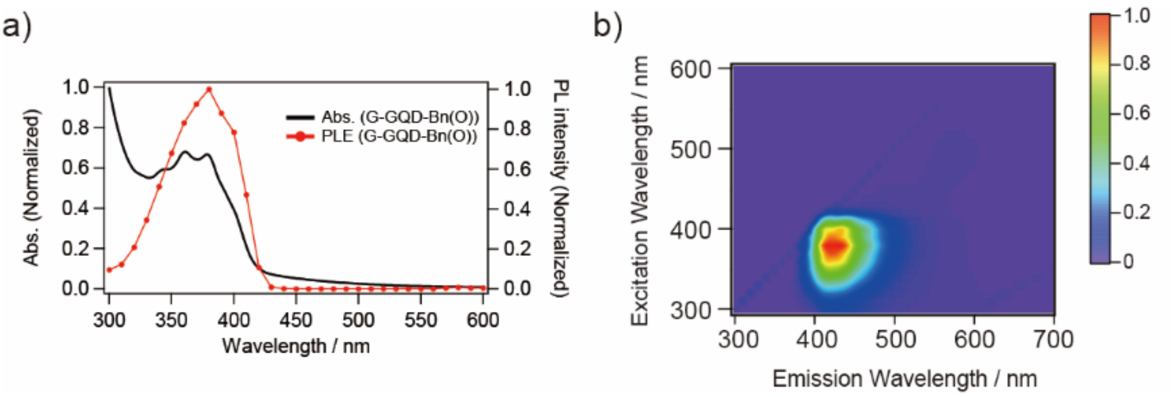

Figure S4. a) Optical absorption spectrum and PL excitation spectrum of the GQD-Bn (O) derived from graphite as the starting material; the emission wavelength is 432 nm. b) PL excitation map for GQD-Bn derived from graphite in toluene. The colour indicates the intensity of the emission normalized at each emission peak.

PL decay of solid-state GQD-Bn.

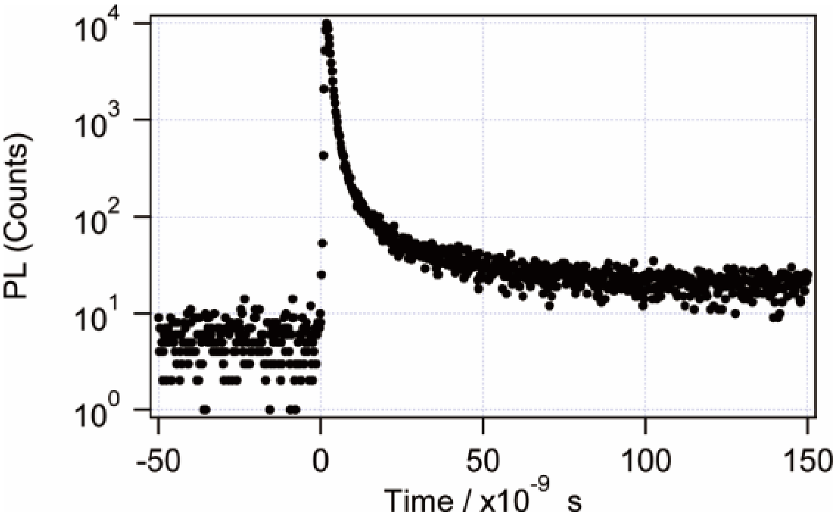

Figure S5. PL decay trace of solid-state GQD-Bn. The vertical axis is plotted on a logarithmic scale. The fluorescence lifetime was counted on a time-correlated single-photon-counting spectrometer (HORIBA, Japan) with an excitation wavelength of 342 nm and a pulse width of 1.1 ns. The repetition rate was 100 kHz.

XPS survey spectrum of GQD-Bn (O).

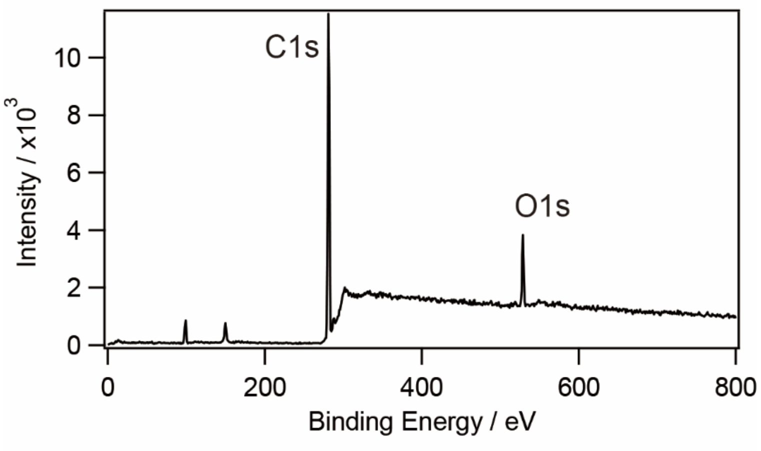

Figure S6. XPS survey spectrum of GQD-Bn (O).
